# Case report: Tracing in parallel the salivary and gut microbiota profiles to assist Larotrectinib anticancer treatment for *NTRK* fusion–positive glioblastoma

**DOI:** 10.3389/fonc.2024.1458990

**Published:** 2024-11-20

**Authors:** Luigia Turco, Rosa Della Monica, Pasqualina Giordano, Mariella Cuomo, Manuele Biazzo, Baptiste Mateu, Raimondo Di Liello, Bruno Daniele, Nicola Normanno, Antonella De Luca, Anna Maria Rachiglio, Carmela Chiaramonte, Francesca Maria Giugliano, Lorenzo Chiariotti, Giuseppe Catapano, Lorena Coretti, Francesca Lembo

**Affiliations:** ^1^ Department of Precision Medicine, University of Campania “Luigi Vanvitelli”, Naples, Italy; ^2^ CEINGE-Advanced Biotechnologies “Franco Salvatore”, Naples, Italy; ^3^ Department of Molecular Medicine and Medical Biotechnologies, University of Naples “Federico II”, Naples, Italy; ^4^ UOC Oncologia Ospedale del Mare, ASL Napoli 1 Centro, Naples, Italy; ^5^ The BioArte, San Gwann, Malta; ^6^ Department of Pharmacy, University of Naples “Federico II”, Naples, Italy; ^7^ Task Force on Microbiome Studies, University of Naples “Federico II”, Naples, Italy; ^8^ Scientific Directorate, IRCCS Istituto Romagnolo per lo Studio dei Tumori (IRST) “Dino Amadori”, Meldola, Italy; ^9^ Cell Biology and Biotherapy Unit, Istituto Nazionale Tumori - IRCCS Fondazione G. Pascale, Naples, Italy; ^10^ UOC Neurochirurgia Ospedale del Mare, ASL Napoli 1 Centro, Naples, Italy; ^11^ UOC Radioterapia Ospedale del Mare, ASL Napoli 1 Centro, Naples, Italy

**Keywords:** glioblastoma multiforme, *NTRK*-gene fusion, oncotherapy, full-length *16S* rRNA gene sequencing, saliva and feces microbiota profiles

## Abstract

Oncotherapy can shape intestinal microbiota, which, in turn, may influence therapy effectiveness. Furthermore, microbiome signatures during treatments can be leveraged for the development of personalised therapeutic protocols in cancer treatment based on the identification of microbiota profiles as prognostic tools. Here, for the first time, the trajectory of gut and salivary microbiota in a patient treated with Larotrectinib, a targeted therapy approved for diagnosed glioblastoma multiforme neurotrophic tyrosine receptor kinase (*NTRK*) gene fusion-positive, has been accurately investigated. We based our analyses on histological diagnosis, genomic and epigenomic profiling of tumour DNA, and faecal and salivary full-length *16S* rRNA gene sequencing. The study clearly evidenced a remodelling of the bacterial communities following 1 month of the NTRK-inhibitor treatment, at both gut and oral levels. We reported a boosting of specific bacteria also described in response to other chemotherapeutic approaches, such as *Enterococcus faecium*, *E. hirae*, *Akkermansia muciniphila*, *Barnesiella intestinihominis*, and *Bacteroides fragilis.* Moreover, several bacterial species were similarly modulated upon Larotrectinib in faecal and saliva samples. Our results suggest a parallel dynamism of microbiota profiles in both body matrices possibly useful to identify microbial biomarkers as contributors to precision medicine in cancer therapies.

## 1 Introduction

Glioblastoma multiforme (GBM) is a primary malignant tumour of the central nervous system (CNS) characterised by poor prognosis. The standardised treatment encompasses neurosurgical resection followed by radio- and adjuvant temozolomide (TMZ) chemotherapy (1). The positive response to TMZ treatment can be predicted by assessing the methylation status of the O6-methylguanine DNA methyltransferase (*MGMT*) promoter region (2). The progress from single-gene to whole-genome DNA methylation analysis has facilitated a more refined subclassification of glioblastomas, introducing novel potential therapeutic targets, such as neurotrophic tyrosine receptor kinase (*NTRK*) fusions. *NTRK* genes exhibit low expression levels in GBM, and their fusions are identified in approximately 0.56%–1.69% of adult patients with GBM, as per The Cancer Genome Atlas database. A recent breakthrough in this context is the approval of Larotrectinib, a tropomyosin receptor kinase (TRK) inhibitor, specifically designed for treating tumours harbouring *NTRK* gene fusions (3, 4).

The CNS has always been considered immune-privileged, finely controlling the adaptive immunity and inflammation. Consequently, the GBM is described as a “cold tumour,” eluding an efficient anticancer immune response (5). The tumour microenvironment limits the immune cell infiltration and becomes immunosuppressive through the secretion of factors like tumour growth factor–β and interleukin-10 (IL-10), promoting regulatory T cell (Treg) generation, and hosting immunosuppressive tumour-associated macrophages, collectively hindering an effective antitumour immune response (6). However, tumour treatment always involves an immune system response. The intestinal microbiota might control not only the gut immune homeostasis but also the inflammatory and/or immune status of secondary lymphoid organs, culminating in shaping the tumour microenvironment and reprogramming the anticancer immune responses (7). Gut microbiome and specific bacteria defined as “oncomicrobiotics” have been shown to have multiple effects on tumour biology, such as the transformation process, tumour progression, and the response to anticancer therapies including immunotherapy (8). Although research specifically focussed on GBM and microbiota is still incipient, there are several human and animal studies in related fields suggesting a potential correlation between microbiota and pharmacological effects of chemotherapies and novel targeted immunotherapies (9–13). While often focussing on other types of cancer, these studies offer important insights that could be relevant for GBM. Gut microbiota has been shown to significantly facilitate immune responses in cancer treatment (7, 9–15). Notably, germ-free mice receiving faecal transplants from responding melanoma patients undergoing anti–programmed cell death 1 protein (PD-1) immunotherapy showed enhanced systemic and antitumour immunity (11). *Enterococcus hirae* and *Barnesiella intestinihominis* ameliorated the immunomodulatory efficacy of cyclophosphamide. In particular, *E. hirae* translocates from the small intestine to secondary lymphoid organs and induces systemic pathogenic T helper 17 (Th17) cell responses and increases intratumoural cytotoxic T lymphocyte (CTL)/Treg cell ratio. *B. intestinihominis* boosts systemic polyfunctional type 1 CD8+ T cells and Th1 cell responses and promotes the intratumour infiltration of interferon-γ–producing γδT cells (7). The important role of the gut microbiota in shaping the immune response may be fundamental for “cold tumour,” potentially boosting the anticancer treatment.

Furthermore, pharmacokinetic modulation operated by gut microbiota might potentiate desirable effects, abrogate efficacy, or release toxic compounds (16). *Mycoplasma hyorhinis*, expressing the enzyme thymidine phosphorylase, increases the effectiveness of the capecitabine metabolite 5-fluoro-5′-deoxyuridine. Bacterial enzymes can also modify the toxic profile of some chemotherapeutics as in the case of the SN-38, Irinotecan’s active metabolite. It can be released by the bacterial β-glucuronidases cleavage into the bowel lumen generating diarrhoea (17). Conversely, chemotherapy induces changes in the diversity and composition of the mucosal and faecal microbiota impacting its functions and profoundly influencing the host’s response to therapy (18). Montassier and colleagues demonstrated profound alterations in gut microbiota after chemotherapy, driving reduced inflammation by the nuclear factor–kappa B pathway modulation and short-chain fatty acids production (19). Although similar studies in GBM are scarce, these findings suggest that the microbiota might similarly influence responses to GBM-targeted therapies like Larotrectinib, potentially offering new ways to improve patient outcomes by modulating the microbiome. Therefore, the gut microbiota could be considered as a marker for predicting treatment response or its modulation as a supplementary treatment option in combination with other cancer treatments.

Gut microbiota is also connected to the oral microbiota in several pathophysiological issues including cancer (20, 21). An association between oral microbiota and the progression of glioma has recently been proposed (22).

In this case study, we carefully defined the composition of the gut and salivary microbiota of a patient diagnosed with GBM *NTRK* fusion–positive at the beginning of Larotrectinib prescription and after 1-month intake. The case report provided here represents the first study associating this novel treatment based on a molecular cancer classification and the patient’s dynamics of microbial communities. This detailed evaluation related to Larotrectinib-treated GBM is rare in the literature, emphasising the unique contribution of our findings. Future studies could build upon this work to further explore how microbiota profiles influence GBM, highlighting the potential for future investigations in this area. The parallel tracking of gut and salivary microbiota profiles could allow a confident screening, possibly useful to identify microbial biomarkers as contributors to precision medicine in cancer therapies.

## 2 Case presentation

A 66-year-old man, after experiencing some neurological symptoms, underwent brain magnetic resonance imaging (MRI) scan, which documented a high-grade glioma. He had a good clinical condition, regular body mass index, and a Karnofsky status of 100%; no comorbidities were reported.

**Figure 1:**
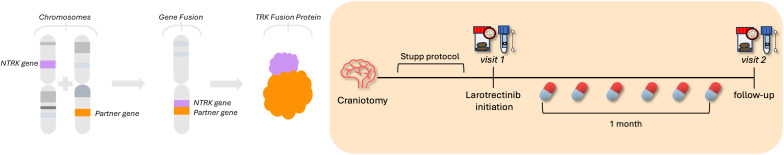
Timeline experiment. A 66-year-old man diagnosed with a high-grade glioma underwent a right temporal craniotomy with a gross total resection: the histological diagnosis was a glioblastoma. The next-generation sequencing fusion panel identified an *NTRK2* gene fusion (left-side figure). After the neurosurgical intervention, the patient received the Stupp protocol, a standard treatment as the first line with radio- and chemotherapy based on temozolomide. Due to neurological symptoms, the patient started a second-line therapy with Larotrectinib. Faecal and salivary samples were collected at the beginning of the treatment and the follow-up was done after 1 month.

After the clinical presentation, the case was discussed by a multidisciplinary CNS neoplasms group. Consequently, the patient underwent a right temporal craniotomy with a gross total resection. The histological diagnosis was a glioblastoma. Epigenomic and genomic profiling of DNA tumour reported an unmethylated status of *MGMT* promoter, a wild-type isocitrate dehydrogenase (IDH); and a methylation profile of Glioblastoma IDH wt. No *BRAF* V600E mutations were detected. The next-generation sequencing fusion panel identified an *NTRK2* gene fusion. After the neurosurgical intervention, the patient received a standard treatment as first line of treatment, with radio- and chemotherapy based on TMZ, according to the Stupp protocol. After one cycle of TMZ post-radiotherapy, there was a progression according to the Response Assessment in Neuro-Oncology (RANO) criteria, both clinically and on the brain MRI. The neuroradiological study described a right temporal recurrence and the appearance of ventricular ependymal and subependymal nodules, resulting in the formation of a tetraventricular hydrocephalus. Clinically, the patient showed apathy and a tendency towards drowsiness. Due to the neurological symptoms, the case was evaluated by the multidisciplinary oncology group, which excluded other neurosurgical indications. At the time of progression, the patient was taking steroids. The patient then started a second-line therapy with oral Larotrectinib according to the standard dose of 100 mg, two times a day, until disease progression or unacceptable toxicity. No toxicity was recorded during the treatment. Faecal and salivary samples were collected at the beginning of the treatment and the follow-up was done after 1 month (**Figure 1**). Since the diagnosis, the patient adhered constantly to an appropriate lifestyle plan. Throughout the study period, no significant changes in diet, medications, or lifestyle occurred that could independently alter microbiota composition. Moreover, we maintained detailed records of the patient’s dietary intake, supplement use, and any additional treatments, ensuring that potential confounding factors were controlled during the analysis. Bacterial DNA was extracted by using the QiAmp Power Faecal Pro DNA Kit (Qiagen). Microbial taxonomy was inferred using a 16S rDNA amplicon sequencing strategy relying on the Oxford Nanopore technology. The *16S* rRNA gene was amplified through PCR reaction targeting V1 to V9 hypervariable regions. Amplicons were then barcoded (enabling sample multiplexing) and adapter-ligated prior to sequencing. MiTRA in-house pipeline was used to infer likely taxonomy.

### 2.1 Post-intervention findings

A single-case study was conducted to examine the changes in bacterial composition before and after 1 month of Larotrectinib treatment, using faecal and salivary microbiota samples. Taxonomy-based methods revealed that cancer treatment affected bacterial profile at gut and oral sites.

At gut level, the treatment induced an enrichment of Firmicutes to the detriment of Bacteroidota and Actinobacteriota. The gut microbiota upon treatment was also characterised by an increase of Synergistota and Verrucomicrobiota, with a fold change of 2.8 and 6.4, respectively (**Supplementary Figure 1**).

**Figure 2:**
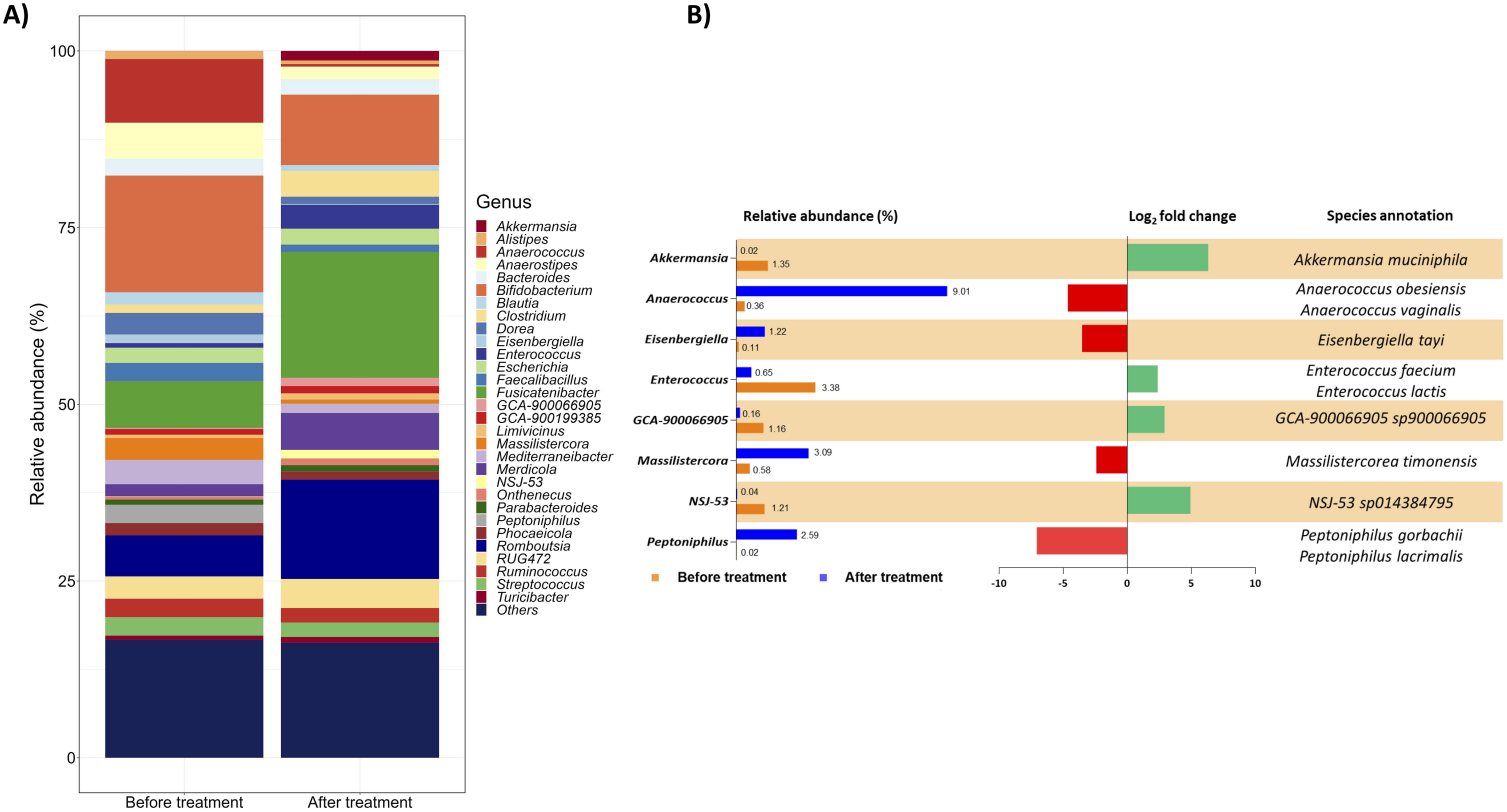
Gut microbial communities’ arrangement at the genus level. **(A)** The taxon bar chart shows the relative abundance (%) of the 30 predominant bacteria at the genus level before and after treatment. **(B)** List of genera selected from the 30 most abundant ones with a log_2_ fold change of at least ±2. For each genus, the percentage of its relative abundance before and after treatment, the log_2_ fold change, and the species annotation are reported. The log_2_ fold change has been calculated as the difference between the log_2_ of the after-treatment relative abundance (%) and the log_2_ of the before-treatment relative abundance (%). The corresponding species were selected considering from all the species identified only those contributing at least 10% to the variation of the corresponding genus. Green and red bars represent a positive and negative fold change to the baseline, respectively.

The bacteria assortment was then studied at the genus level. Focussing on the thirty most abundant genera, the treatment notably reduced the genera *Alistipes*, *Anaerostipes*, *Bifidobacterium*, *Blautia*, *Dorea*, *Faecalibacillus*, and *Mediterraneibacter* and increased *Clostridium*, *Fusicatenibacter*, *Merdicola*, and *Romboutsia* (**Figure 2A**). Moreover, considering a log_2_ fold change threshold of ±2, the genera *Akkermansia*, *Anaerococcus*, *Eisenbergiella*, *Enterococcus*, *GCA-900066905*, *Massilistercora*, *NSJ-53*, and *Peptoniphilus* were particularly impacted by a 1-month administration of the targeted therapy (**Figure 2B** also details annotations for relevant species selected considering, from all the species identified, only those contributing at least 10% to the variation of the corresponding genus).

**Figure 3:**
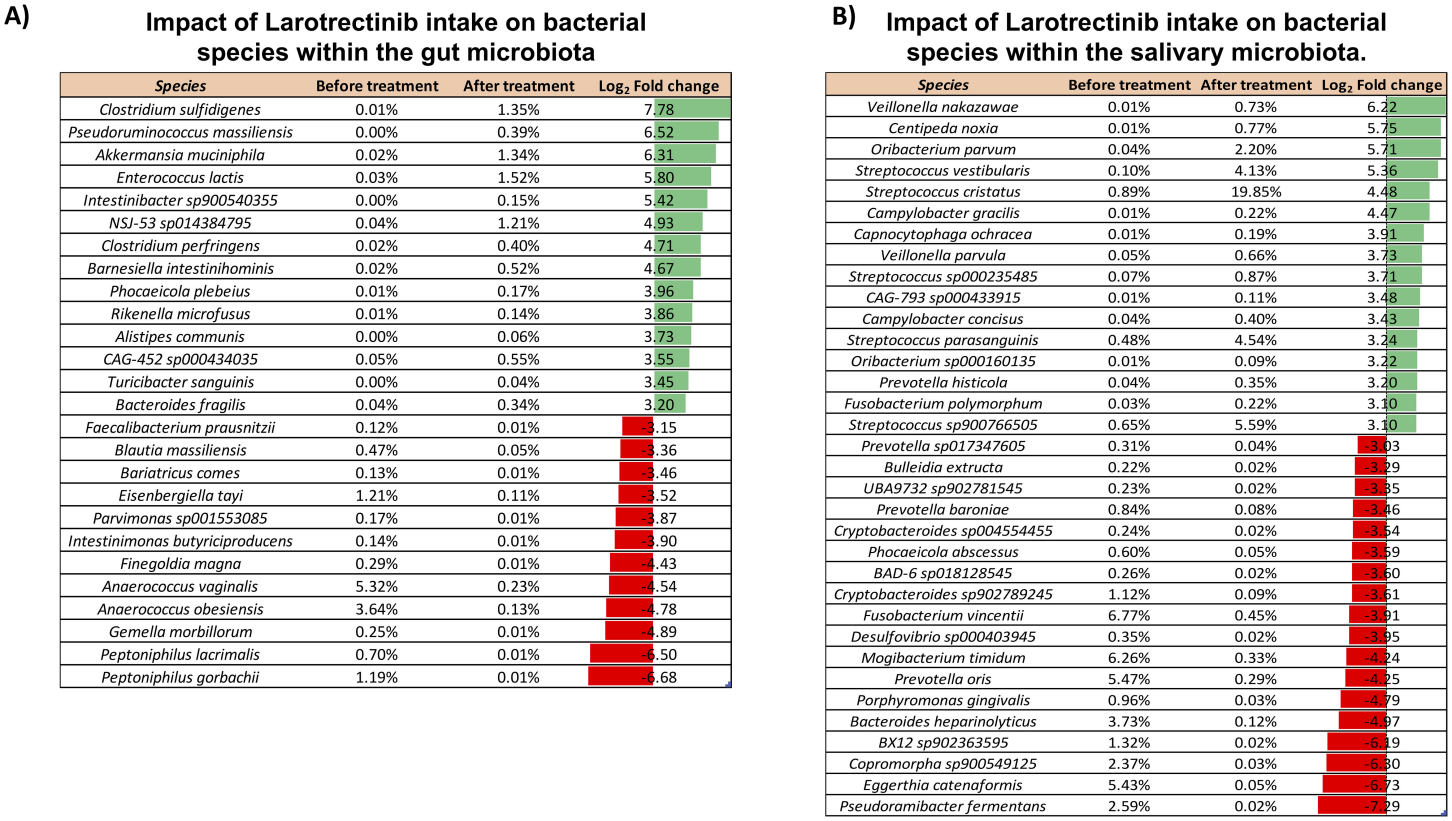
Impact of Larotrectinib intake on bacterial species within the gut and salivary microbiota. **(A)** Subset of species sorted out from the 484 overall identified in the gut microbiota by applying a cutoff criterion of a log_2_ fold change of at least ±3. **(B)** Subset of species sorted out from the 265 overall identified in the salivary microbiota by applying a cutoff criterion of a log_2_ fold change of at least ±2. For each species featured, the percentage of its relative abundance before and after treatment and the log_2_ fold change are presented. The log_2_ fold change has been calculated as the difference between the log_2_ of the after-treatment relative abundance (%) and the log_2_ of the before-treatment relative abundance (%). Green and red bars represent a positive and negative fold change to the baseline, respectively.

To specifically investigate the microbial composition at the species level, we thoroughly curated a subset from the total pool of 484 species (**Supplementary Dataset**). Applying a log_2_ fold change threshold of ±3, we pinpointed species exhibiting substantial alterations upon the Larotrectinib treatment, including those with low abundance. This analysis revealed a noteworthy rise in the abundance of *Pseudoruminococcus massiliensis*, *Intestinibacter sp900540355*, *Barnesiella intestinihominis*, *Phocaeicola plebeius*, *Rikenella microfusus*, *Alistipes communis*, *CAG-452 sp000434035*, *Turicibacter sanguinis*, and *Bacteroides fragilis*. Conversely, we observed a reduction in the levels of *Faecalibacterium prausnitzii*, *Bariatricus comes*, *Parvimonas sp001553085*, *Intestinimonas butyriciproducens*, *Finegoldia magna*, and *Gemella morbillorum* (**Figure 3A**).

The comprehensive compositional analysis was then conducted to investigate the salivary microbiota response to the oral administration of Larotrectinib. At phylum level, aside from the Actinobacteriota and Proteobacteria, all other phyla exhibited a noteworthy twofold increase or decrease, relative to their pre-treatment levels (**Supplementary Figure 2**). Investigating the impact of the cancer treatment on species profiles, we found that several species showed significant changes when a log_2_ fold change threshold of ±3 was applied. Among these, species belonging to the *Streptococcus* genus were strongly increased upon Larotrectinib treatment, whereas *Fusobacterium vincentii*, *Mogibacterium timidum*, *Prevotella oris*, and *Eggerthia catenaformis* were drastically reduced (**Figure 3B**).

**Figure 4:**
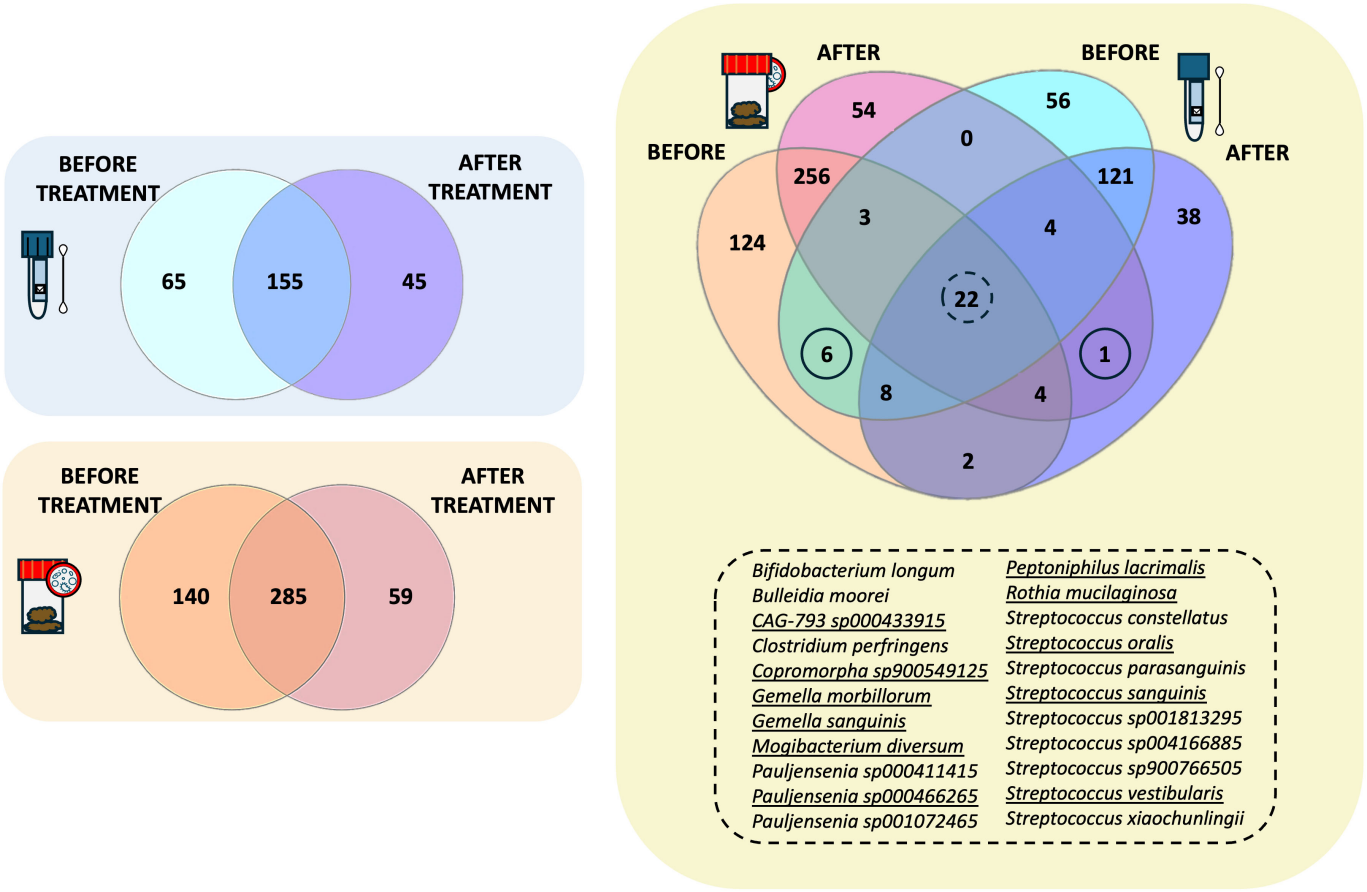
Intersectional analysis of the gut and salivary microbiota. Venn diagrams showing the species shared before and after treatment at the gut level and within the salivary microbiota, independently. On the right side, the Venn diagram present the shared species between the gut and the salivary microbiota at the two different experimental time points.

Venn diagram analysis was then applied to highlight the species replacement at both body matrices throughout the treatment (**Figure 4**). The intestinal microbiota consisted of 484 different species, 59 of which were potentially related to Larotrectinib intake, whereas 140 species characterised the gut microbiota before treatment (**Figure 4**). In the salivary microbiota, we identified a total of 265 distinct bacterial species (**Supplementary Dataset**); 155/265 species were common to the two experimental time points and 45 species specifically appeared after 1 month of cancer treatment (**Figure 4**).

Exploring the compositional overlap between the gut and the salivary microbiota, we found 39 and 31 species shared before and after treatment, respectively (**Figure 4**). Among these, 22 species were consistently shared between the two microbiotas at both experimental time points (**Figure 4**, list of shared species). Of note, 11 of them exhibited the same trend in both matrices, suggesting a susceptibility to Larotrectinib treatment. Six species were exclusively shared between the gut and salivary microbiota before treatment, including *Fimenecus sp004556705*, *CAG-313 sp900539265*, *Lawsonella clevelandensis*, *BX12 sp014333425*, *Prevotella sp000479005*, and *S5-A14a sp000758905*, whereas *Streptococcus sp001556435* was the sole species harboured at both gut and oral sites after 1 month intake of Larotrectinib (**Figure 4**).

Therefore, a remodelling of the bacterial communities was observed following 1 month of the NRTK-inhibitor treatment at gut and oral levels. By tracking the changes in microbial profiles at both body matrices, we found definite species similarly regulated upon Larotrectinib, which suggests a parallel dynamism of both microbiotas.

## 3 Discussion

This case study represents the first report of gut and salivary microbiota monitoring in a patient treated with Larotrectinib, a targeted therapy approved for diagnosed GBM *NTRK* fusion–positive. We are aware that the direct effect and mechanism of Larotrectinib on microbial populations should be further explored. We report here a remarkable remodelling of gut and salivary microbiotas, in which the effect should be considered in the context of many other factors, such as site and stage of the tumour, genetic tumour signature, and inflammatory surrounding environment, to assess a plausible role for microbes-host interaction in the cancer therapy outcomes. At the gut level, in the new microbial setting induced by Larotrectinib, we highlighted the enrichment of the *Akkermansia* (*A. muciniphila*) and *Enterococcus* (*E. faecium*, *E. lactis* and *E. hirae*) genera and of the *Barnesiella intestinihominis* and *Bacteroides fragilis* species. Interestingly, the boosting of these bacteria has also been described in response to other oncotherapeutic approaches (7, 9, 10, 12). *E. faecium*, *E. hirae*, and *A. muciniphila* have been found increased in patients with cancer undergoing anti–PD-1 therapy (12). Routy et al. found that oral administration of *A. muciniphila* alone or in combination with *E. hirae* in mice, following faecal microbiota transplantation from non-responder patients to anti–PD-1 therapy, was able to restore antitumour activity (12). The immunological changes induced by *A. muciniphila* and *E. hirae* in mice included the promotion of IL-12 secretion by dendritic cells and the accumulation of CCR9+ CXCR3+ CD4+ T cells in the tumour microenvironment. To the same extent, *E. faecium*, *B. intestinihominis*, and *B. fragilis* have been demonstrated to enhance anticancer therapy efficacy and tumour response to immunomodulation (7, 9, 10, 13). *Bacteroides fragilis*, which presents a log_2_ fold change of 3.20 after treatment, is known to enhance the antitumour effect of the immune checkpoint inhibitors, increasing the efficacy of CTLA-4 blockade, and was found to enhance the antitumour efficacy of oxaliplatin (9, 10). *E. faecium* was reported to boost the anti–PD-1 therapy response in patients suffering from metastatic melanoma (13). Among the species that we found boosted after 1-month intake of Larotrectinib, *Alistipes communis* could participate in supporting immunity. Several species belonging to the *Alistipes* genus have been reported as associated with an optimal cancer immune response (15, 23). These findings suggest a potential role for specific bacteria, which may support Larotrectinib’s therapeutic effects by fostering a more favourable immune environment.

Wen et al. demonstrated the association between oral microbiota composition and glioma progression (22). The phylum Patescibacteria was found inversely correlated with the disease progression, presenting a diagnostic and prognostic determinant for glioma malignancy (22). Accordingly, the patient involved in this case report showed an increase in Patescibacteria levels after 1 month of treatment. Additionally, *Capnocytophaga sputigena*, previously reported as inversely associated with glioma grades (22), was increased in the patient’s saliva upon Larotrectinib treatment. We found a decrease in *Porphyromonas* genus upon treatment, for which high abundance seems to be characteristic of higher-grade gliomas compared to healthy controls (22). Additionally, *Prevotella* and *Mogibacterium* species were associated with a higher risk of colorectal cancer (24), and increased levels of *Fusobacterium*, *Prevotella*, and *Mogibacterium* have been linked to overall cancer risk, whereas *Streptococcus* is inversely related (25). Accordingly, Larotrectinib treatment led to a reduction in species belonging to *Fusobacterium*, *Prevotella*, and *Mogibacterium* genera, with an increase in streptococci, suggesting a potentially beneficial shift in the microbiota composition in response to the therapy.

The inter-organ microbial network is emerging as an important regulator in physiological functions and pathological processes. It is known that the oral microbiota affects the gut microbiota shape and translocates to the intestine when the oral-gut barrier is impaired (26). In the present study, the Venn diagram comparative analysis identified 39 and 31 bacterial species shared by the oral and intestinal microbiota before and after treatment, respectively. Among the species identified, those belonging to the genera *Bifidobacterium*, *Prevotella*, *Streptococcus*, and *Veillonella* are normally found in both oral and intestinal microbiota (27–29). These findings confirm the interconnection between the oral and gut microbiota, emphasising the need to investigate whether the oral microbiota might serve as a more accessible sampling site for convenient and widespread screening. Moreover, we found a parallel reduction of *Gemella morbillorum*, *Peptoniphilus lacrimalis*, and *Rothia mucillaginosa* after Larotrectinib treatment. *G. morbillorum*, *Peptoniphilus* spp., and *R. mucillaginosa* have been linked to bacteremia in cancer and immunosuppressed subjects and could negatively influence chemotherapy due to infection risk (30–32). These results may support the hypothesis that bacterial reassortment 1 month after cancer treatment shifts towards a more favourable immune environment.

Furthermore, we identified seven species (*Fimenecus sp004556705*, *CAG-313 sp900539265*, *Lawsonella clevelandensis*, *BX12 sp014333425*, *Prevotella sp000479005*, *S5-A14a sp000758905*, and *Streptococcus sp001556435*) potentially eligible as predictive of response to Larotrectinib. This case report aims to provide a first link between the GBM treatment and microbiota modulation to pursue the introduction of microbiota-based interventions in the personalised medicine of patients with GBM. Further studies would be valuable to confirm whether this microbial shift persists throughout the 1-month period and in the long term, both after completing therapy with Larotrectinib or in case of recurrence. Monitoring specific bacterial species linked to improved outcomes could help tailor treatments like Larotrectinib. Additionally, microbiota modulation (e.g., probiotics or prebiotics) could be used to enhance therapy efficacy, optimising the tumour microenvironment and improving patient outcomes.

## 4 Conclusion

The case report herein presented attempted to investigate, for the first time, the composition of the gut and salivary microbiota in a patient treated with Larotrectinib, a targeted therapy approved for the treatment of very rare cases of GBM molecularly classified as fusion-positive *NTRK* gene. Our findings suggest that certain bacterial taxa, namely, *A. muciniphila*, *E. faecium*, *E. hirae*, *B. intestinominis*, and *B. fragilis*, may contribute to the immune landscape modulation during cancer treatment (7, 9, 10, 12, 13). Conversely, bacteria showing a relative decline upon Larotrectinib, such as *G. morbillorum* and *R. mucillaginosa*, have been previously reported to potentially hinder chemotherapy efficacy (30–32). While our study was not specifically designed to confirm these mechanisms, our data align with existing literature, indicating that the microbiome could play a critical role in shaping immune responses to assist cancer therapy. The parallel tracking of both microbiotas intended to emphasise the importance of establishing an overlap between the gut and salivary microbiota during anticancer therapies to make microbiota analysis more accessible. Even though case studies based on a single subject have inherent limitations, they highlight extremely unique medical cases.

Our findings imply that a more extensive study, covering a larger number of cases, could potentially identify 1) microbial biomarkers predictive of the oncotherapeutic outcomes; 2) bacterial species concomitantly modulated in gut and saliva upon anticancer therapies; 3) the eligibility of fast and precise techniques for microbial biomarkers detection such as the full-length *16S* rRNA gene sequencing; and 4) the combination of cancer therapy with a microbiota-based intervention as an adjuvant.

## Data Availability

The sequences reported in this study are deposited in the ‘European Nucleotide Archive’ under the accession number PRJEB81154.
