# Supplementary material for: Case report: Tracing in parallel the salivary and gut microbiota profiles to assist Larotrectinib anticancer treatment for NTRK fusion–positive glioblastoma
Source: Front Oncol. 2024 Nov 20;14:1458990. doi: 10.3389/fonc.2024.1458990 (PMC11614819; doi:10.3389/fonc.2024.1458990)
Supplement: Supplementary file 1 [file DataSheet1.docx]

Supplementary Material

# Supplementary Figures


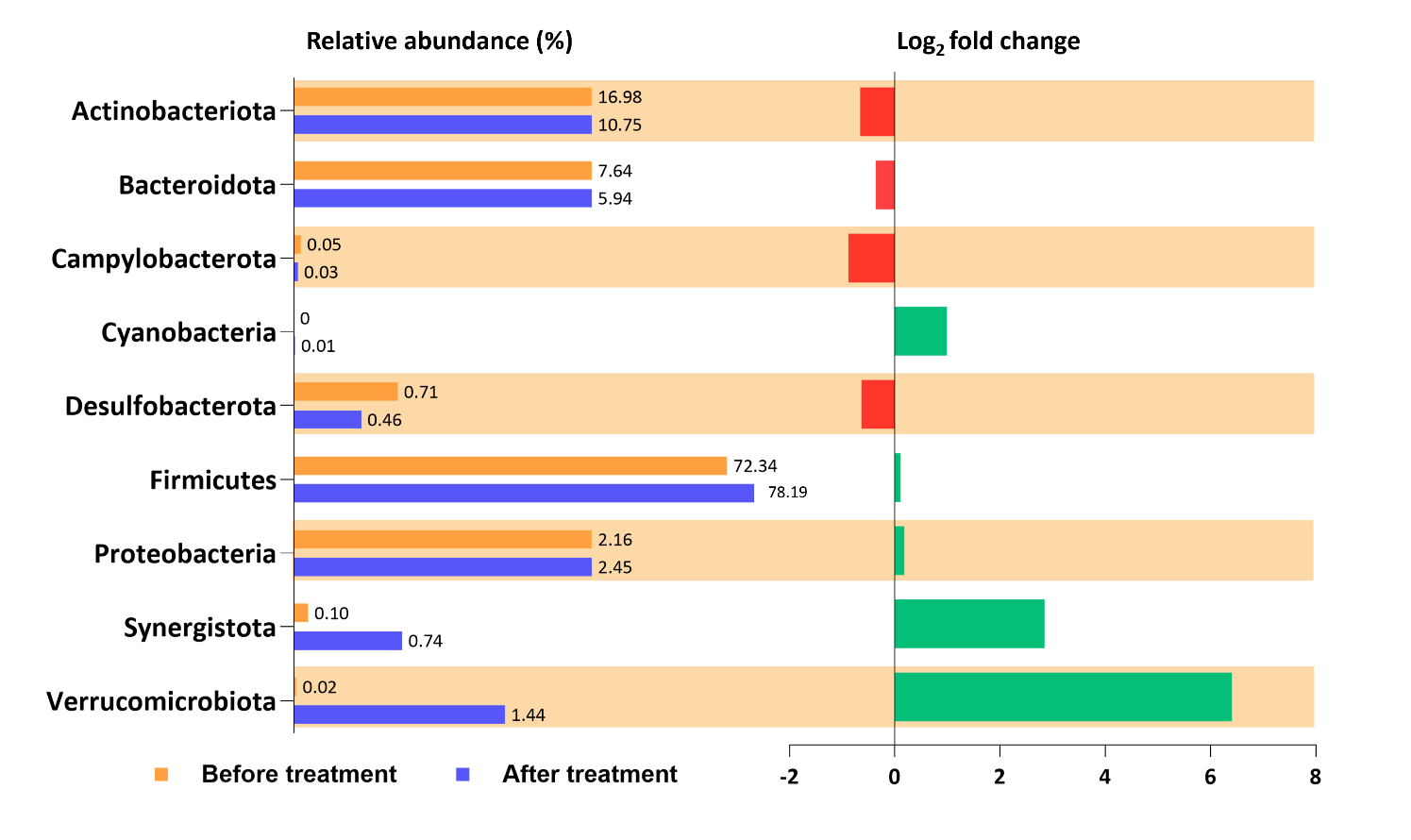


**Supplementary Figure 1.** **Gut microbiota composition at the phylum level.** For each phylum, the percentage of its relative abundance before and after treatment and the log_2_ fold change were reported. The log_2_ fold change has been calculated as the difference between the log_2_ of the after-treatment relative abundance (%) and the log_2_ of the before-treatment relative abundance (%). Green and red bars represent a positive and negative fold change with respect to the baseline, respectively.


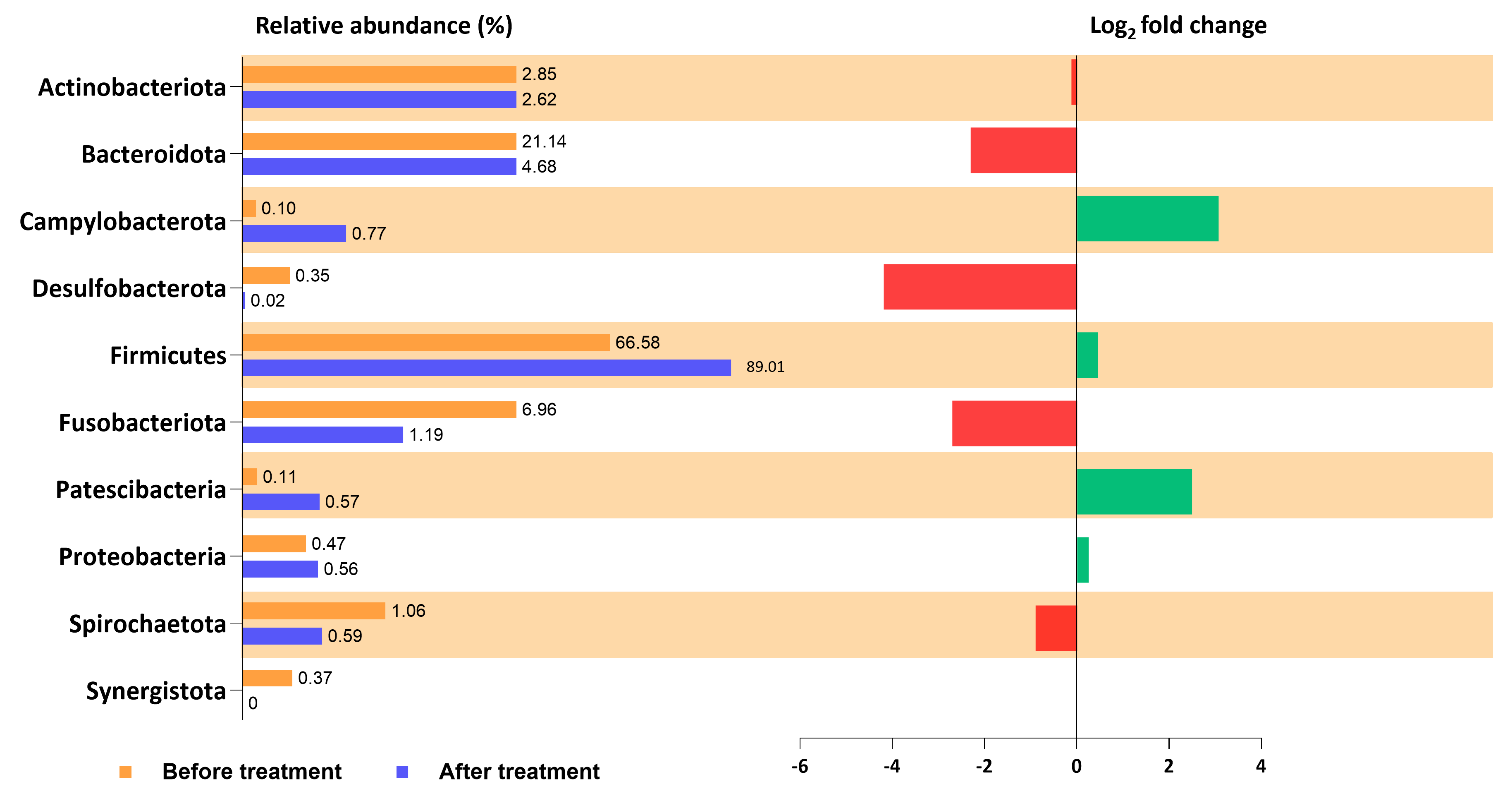


**Supplementary Figure 2.** **The composition of the salivary microbiota at the phylum level.** For each phylum, the percentage of its relative abundance before and after treatment and the log_2_ fold change were reported. The log_2_ fold change has been calculated as the difference between the log_2_ of the after-treatment relative abundance (%) and the log_2_ of the before-treatment relative abundance (%). Green and red bars represent a positive and negative fold change with respect to the baseline, respectively.
